# First-Line Autologous Stem Cell Transplantation for Mantle Cell Lymphoma: A Systematic Analysis and Treatment Recommendation

**DOI:** 10.3389/fonc.2022.881346

**Published:** 2022-05-11

**Authors:** Hailing Liu, Xiao Shi, Huizi Fang, Lei Cao, Yi Miao, Xiaoli Zhao, Wei Wu, Wei Xu, Jianyong Li, Lei Fan

**Affiliations:** ^1^ Department of Hematology, Jiangsu Province Hospital and Nanjing Medical University First Affiliated Hospital, Nanjing, China; ^2^ Nanjing Pukou District Central Hospital, Pukou Branch of Jiangsu Province Hospital, Pukou CLL Center, Nanjing, China; ^3^ Jiangsu Key Lab of Cancer Biomarkers, Prevention and Treatment, Collaborative Innovation Center for Personalized Cancer Medicine, Nanjing Medical University, Nanjing, China

**Keywords:** mantle cell lymphoma, autologous stem cell transplantation, rituximab, meta-analysis, treatment

## Abstract

**Background:**

In the era of immunotherapy, autologous stem cell transplantation (ASCT) in first-line therapy in patients with mantle cell lymphoma (MCL) has been a controversial topic. This report aimed to explore the association between ASCT and MCL survival through a systematic review with meta-analysis.

**Methods:**

We performed a systematic search of original articles published from inception to September 2021 using PubMed, MEDLINE, Embase, and Cochrane Library databases.

**Results:**

We included studies that compared ASCT with non-ASCT consolidation in newly diagnosed transplant-eligible MCL. The endpoints were progression-free survival (PFS) and overall survival (OS). There were seven eligible studies (one randomized clinical trial, one prospective cohort study, and five observational studies) published between 2012 and 2021, in which the total number of participants was 3,271. In the non-intensive induction subgroup, patients with ASCT experienced a significant PFS but no OS benefit compared with those without ASCT. In the intensive induction subgroup, the PFS benefit from ASCT still existed but largely attenuated; no OS benefit was observed though only one study was suitable for evaluation. When compared to the rituximab maintenance arm, ASCT had a worse PFS and OS.

**Conclusions:**

In the rituximab plus HiDAC era, the benefit of ASCT as a component of first-line treatment has been weakened. First-line maintenance strategy instead of ASCT seems worth exploring .

## Introduction

Mantle cell lymphoma (MCL) is an aggressive and incurable non-Hodgkin lymphoma, with an increasing annual incidence of 0.8 per 100,000 population in Western countries (1). Survival for MCL patients (pts) has historically been very dismal, with median overall survival (OS) in the range of 3–5 years (2, 3). Front-line consolidative ASCT was first recommended to treat young MCL pts to prolong survival, whose support was from evidence in the pre-rituximab era (4). However, some scholars showed the benefit of ASCT as a component of first-line treatment was weakened in the rituximab era (5–7). Moreover, several studies demonstrated the survival superiority of intensive induction regimens such as the Nordic regimen (8), DHAP alternating with R-CHOP (9), and R-hyper-CVAD with methotrexate and high-dose cytarabine (HiDAC) (10, 11) compared with historical controls. Chemo-immunotherapy containing rituximab and HiDAC followed by ASCT started to become a widely accepted standard for newly diagnosed transplant-eligible MCL (12), but notably, ASCT has once more not been proven to be a necessary protocol with survival benefit in the rituximab plus HiDAC era. In the study by LaCasce et al. (13), R-hyper-CVAD with or without ASCT had similar disease-specific outcomes, firstly raising the question about the consolidation role of ASCT under the premise of using rituximab and HiDAC. Even the newest follow-up data from the only randomized controlled trial (RCT) supporting the value of ASCT consolidation reported that ASCT failed to prolong progression-free survival (PFS) and OS after a median follow-up of 14 years (14). This controversy is evident in the current practice, but no well-designed prospective clinical trials have resolved it. It would be helpful to combine data from available studies to get a clearer view of the therapeutic value of ASCT in MCL. We therefore conducted a systematic review and meta-analysis on the treatment efficacy of ASCT for newly diagnosed transplant-eligible MCL pts.

## Materials and Methods

### Search Strategy and Study Selection

We systematically searched PubMed, Medline, Embase, and Cochrane Library databases from inception to August 2021. We combined subject words with free words for retrieval and adjusted according to the characteristics of different databases without any restriction on gender, ethnicity, and languages to reduce bias. **Supplemental File 1** shows this in more detail. Two investigators independently assessed RCTs using the Jadad scale and non-RCTs using the Newcastle-Ottawa Scale (NOS). The populations, interventions, comparisons, outcomes, and study designs (PICOS) considered for review are listed in **Table 1**. The co-primary variables were OS, defined as the time from random assignment to death from any cause, and PFS, defined as the time from randomization to documented disease progression or death. Three investigators independently screened the studies and solved any disagreements regarding trial selection through discussion. The search process is depicted in the Preferred Reporting Items for Systematic Reviews and Meta-Analyses (PRISMA) flowchart, outlining the numbers of identified and excluded records and the final number of included trials.

**Table 1:** Population, interventions, comparisons, outcomes, and study design (PICOS) criteria for study.

Criteria	Definition
Population	ASCT-eligible patients with newly diagnosed MCL
Interventions	Rituximab-containing induction regimens followed by ASCT
Comparisons	Rituximab-containing induction regimens followed by non-ASCT strategies, such as IFNα maintenance, rituximab maintenance, and observation
Outcomes	Overall survival; progression-free survival
Study Design	RCTs (Jadad scores ≥3 points); prospective or retrospective observational cohort studies, and case–control studies (NOS scores ≥7 points)

MCL, mantle cell lymphoma; ASCT, autologous stem cell transplantation; RCT, randomized controlled trial; NOS, the Newcastle-Ottawa Scale.

### Data Extraction and Statistical Analysis

Study characteristics, including first author, year of publication, data sources, study type, recruitment interval, the number of ASCT-eligible pts, and median follow-up, were extracted. The principal data for analysis derived from included studies were the log hazard ratio (HR) and its standard error or the information to estimate them, such as an HR and its 95% confidence interval (CI) or Kaplan–Meier (KM) plots. When raw data were not available, we used Adobe Photoshop to process the KM curve pictures and Engauge Digitizer 11.1 software (http://digitizer.sourceforge.net) to extract survival data, then pooled the effect estimates together using the fixed and random-effects model. Statistical heterogeneity was tested by Cochran’s *Q* (*χ* ^2^) (*p* < 0.10) and quantified by *I* ^2^, where *I* ^2^ of 0%–25% indicates no or mild heterogeneity, 25%–50% indicates moderate heterogeneity, 50%–75% indicates high heterogeneity, and 75%–100% indicates extreme heterogeneity. Furthermore, we conducted subgroup analyses and sensitivity analyses to identify the possible causes of substantial heterogeneity. A risk-of-bias assessment was also planned but was not applicable due to the small number of included trials.

## Results

### Screening Process

**Figure 1:**
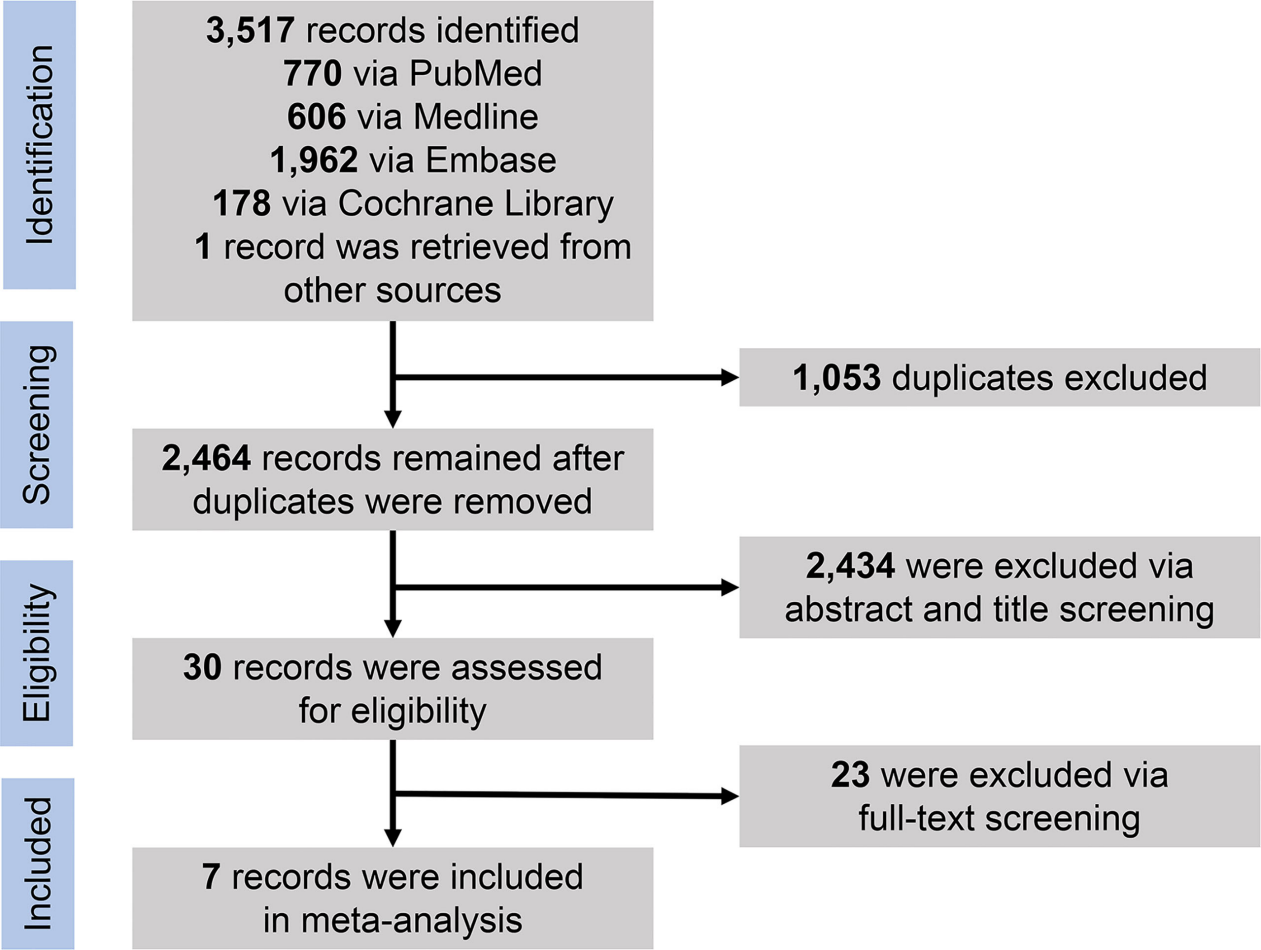
PRISMA flowchart for selection of studies. PRISMA, Preferred Reporting Items for Systematic Reviews and Meta-Analyses.

Through the initial search of four databases (PubMed, Medline, Embase, and Cochrane Library databases) and hand-searching relevant bibliographies, we identified 3,517 records and then excluded 1,053 duplicates through literature manager software. Based on title/abstract content, three authors independently reviewed and excluded 2,434 references that did not satisfy the selection criteria. After reviewing the full text of the remaining 30 records, we excluded 23 for the following reasons: same data source (*n* = 6), no available data on results (*n* = 11), and low quality (*n* = 6). Finally, seven studies published between 2012 and 2021 met our inclusion criteria, including one RCT, one prospective cohort study, and five observational studies. **Figure 1** shows the PRISMA diagram.

### General Study Characteristics

**Table 2** shows the general characteristics of included studies (5, 13–18). The final datasets contained 3,271 pts, with a recruitment interval of 1996 to 2019 and a median age ranged from 55 to 62 years. The shortest median follow-up was 33 months, and the longest was 168 months. All induction regimens included rituximab and were classified into intensive and non-intensive induction groups. The former involved HiDAC-containing regimens, R-hyper-CVAD, and R-maxi-CHOP, while the latter include R-CHOP, BR, VcR-CVAD (5), and so on. Post-induction strategies consisted of ASCT consolidation, IFNα maintenance, rituximab maintenance (RM), and observation.

**Table 2:** Characteristics of included studies.

Author and Year	Data sources	Study type	Interval	Number of ASCT-eligible pts	ASCT arm *n* (%)	Non-ASCT arm *n* (%)	Median follow-up
LaCasce AS (13)	The NCCN NHL Database (7 NCCN centers)	Non-RCT	2000–2008	167	55 (32.9)	112 (67.1)	33 months
Chang JE (5)	The Eastern Cooperative Oncology Group (multicenter)	Non-RCT	2007–2008	66	22 (33.3)	44 (66.7)	54 months
Abrahamsson A (15)	Swedish and Danish lymphoma registries (multicenters)	Non-RCT	2000–2011	1,143	273 (23.9)	870 (76.1)	107 months
Gerson JN (16)	25 North American academic centers	Non-RCT	2000–2015	1,029	657 (63.8)	372 (36.2)	76 months
Wang YH (17)	3 Taiwan academic centers	Non-RCT	2006–2019	97	41 (42.3)	56 (57.7)	60.5 months
Karmali R (18)	12 US academic centers	Non-RCT	2000–2015	595	350 (58.8)	245 (41.2)	48 months
Zoellner AK (14)	The European MCL Network (121 institutions)	RCT	1996–2004	174	93 (53.4)	81 (46.6)	168 months

ASCT, autologous stem cell transplantation; MCL, mantle cell lymphoma; NCCN, National Comprehensive Cancer Network; NHL, non-Hodgkin lymphoma; RCT, randomized controlled trial.

### Quality Assessment and Bias Risk

**Figure 2:**
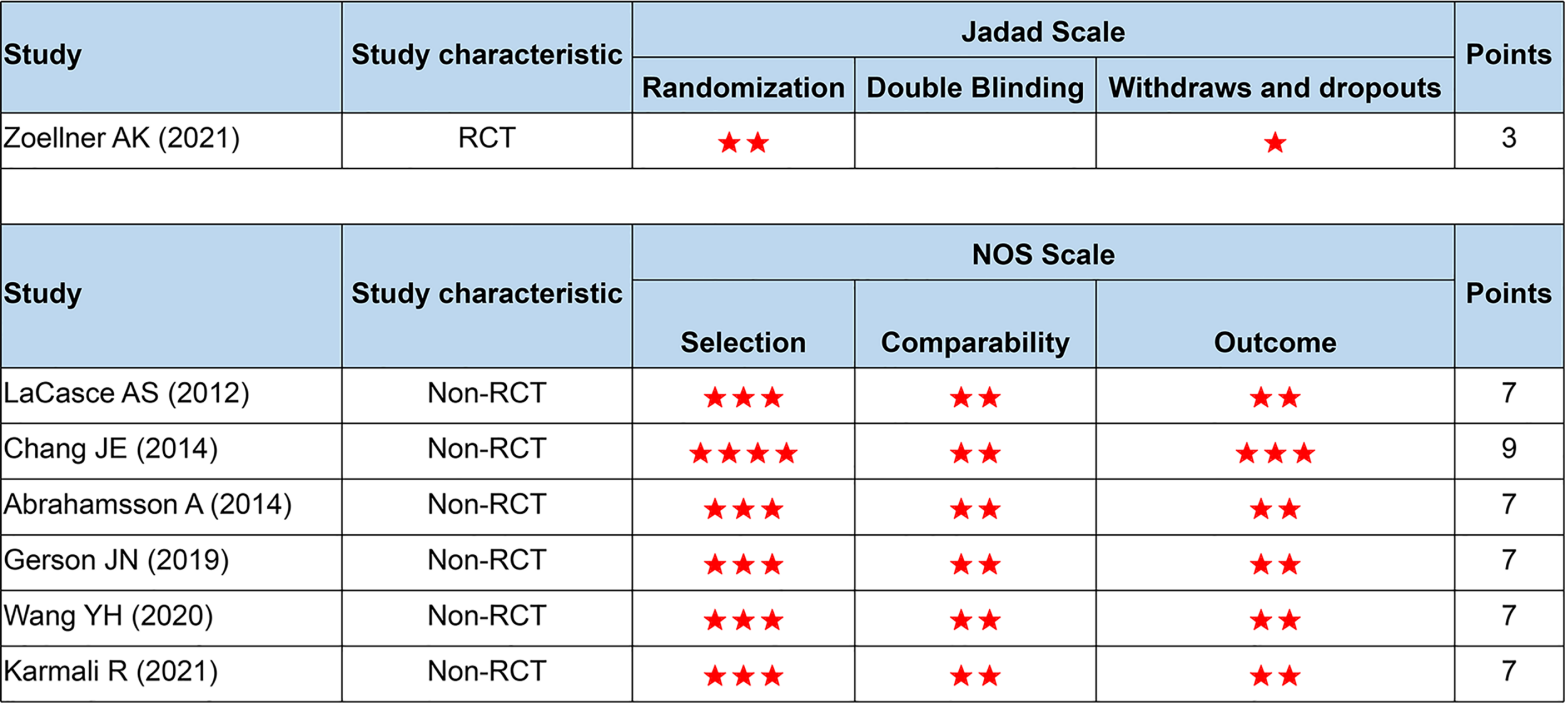
Quality assessment based on the Jadad and NOS scale. NOS, the Newcastle-Ottawa Scale; RCT, randomized controlled trials. Number of asterisks denotes scores.

As in **Figure 2**, all studies were of high quality and low bias risk (one RCT scored 3 points using the Jadad scale, and six non-RCTs scored 7–9 points using the NOS scale). Because the number of included studies was less than 10, we did not perform the funnel plot.

### Effect of ASCT on PFS

**Figure 3:**
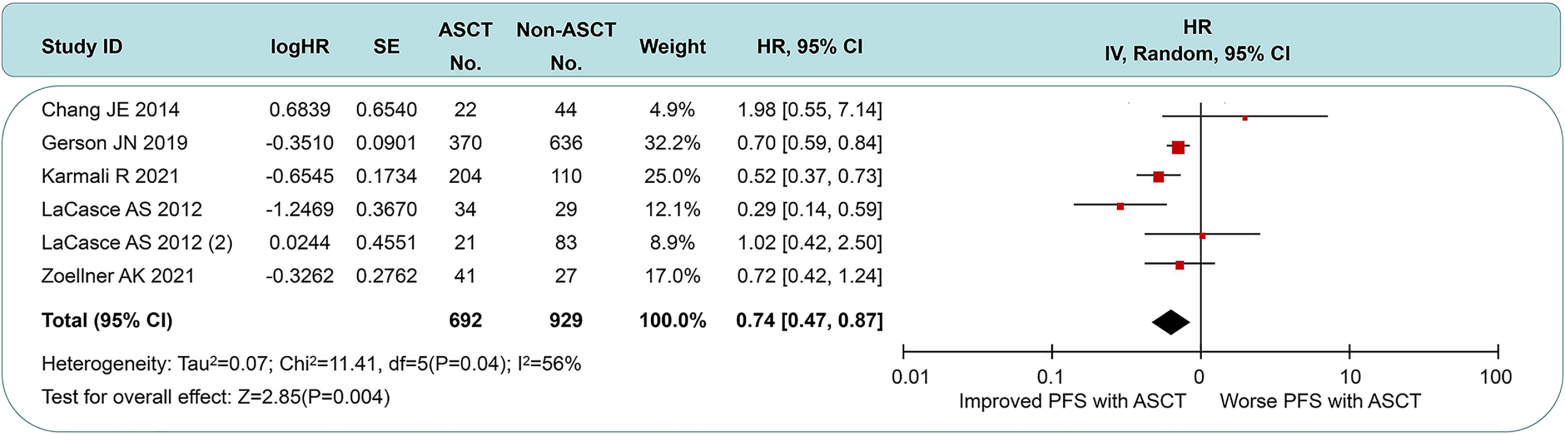
Forest plot and meta-analysis of progression-free survival between ASCT and non-ASCT group in MCL patients. ASCT, autologous stem cell transplantation; MCL, mantle cell lymphoma.

Five of the seven studies were available for HR calculation of PFS between ASCT and non-ASCT arms. Due to the lack of the comparison of overall data in the study by LaCasce et al. (13), we exhibited two data subsets according to the original article. Compared with the non-ASCT arm, pts undergoing ASCT experienced a significant PFS improvement (HR = 0.64, 95% CI 0.47 to 0.87). However, moderate heterogeneity existed among these trial results (*I* ^2^ = 56%, *p* = 0.04). Among those, two studies (rows 1 and 4) showed minimal 95% CI overlap. After eliminating the above data, there was little heterogeneity between the remaining studies (*I* ^2^ = 11%, *p* = 0.34), whose sensitivity analysis demonstrated stable outcomes across the *I* ^2^ value (range, 0% to 40%). These results led us to postulate that this heterogeneity could originate from the intensity of first-line induction protocols and the choice of post-induction strategies. See **Figure 3** for details.

**Figure 4:**
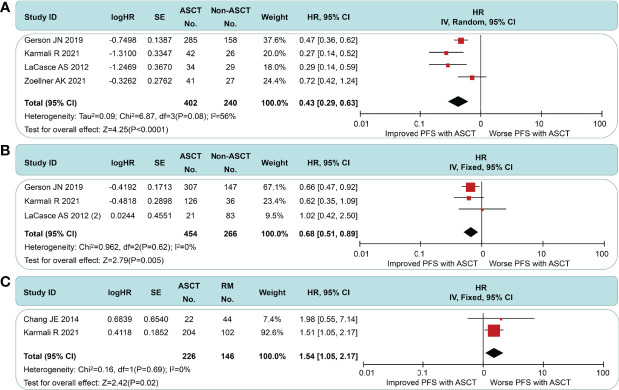
Meta-analysis results of progression-free survival: **(A)** Subgroup analysis for patients with non-intensive induction. **(B)** Subgroup analysis for patients with intensive induction. **(C)** Survival analysis between patients undergoing autologous stem cell transplantation and rituximab.

In the non-intensive induction subgroup, pts undergoing ASCT yield a better PFS (HR = 0.43, 95% CI 0.29 to 0.63) than the non-ASCT arm, but the long-term outcomes of Zoellner et al. displayed marked heterogeneity (**Figure 4A**). If we had chosen to use the short-interval follow-up data (logHR = −0.499, SE = 0.3291, extracted by software) in Dreyling et al.’s paper (4), the *I* ^2^ value decreased from 56% to 46%. We speculate that this heterogeneity may stem from the length of follow-up and the addition of rituximab. In the intensive induction subgroup, a PFS benefit was still found in MCL pts treated with ASCT compared to pts without ASCT (HR = 0.58, 95% CI 0.51 to 0.89), but this was less significant than the non-intensive induction subgroup (**Figure 4B**). There was no evidence of heterogeneity (*I* ^2^ = 0%, *p* = 0.61). As in **Figure 4C**, the ASCT arm exhibited a worse PFS than the rituximab arm (HR 1.54, 95% CI 1.09 to 2.18). Despite the smaller sample size, there was high homogeneity between the groups (*I* ^2^ = 0%, *p* = 0.69).

### Effect of ASCT on OS

**Figure 5:**
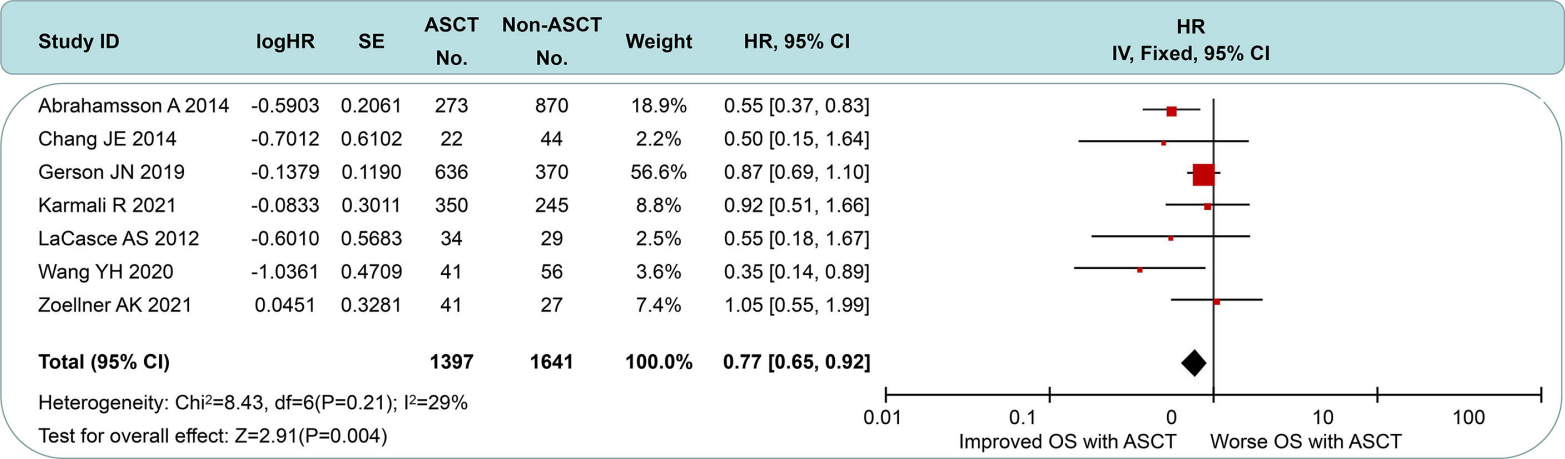
Forest plot and meta-analysis of overall survival between the ASCT and non-ASCT group in MCL patients. ASCT, autologous stem cell transplantation; MCL, mantle cell lymphoma.

**Figure 6:**
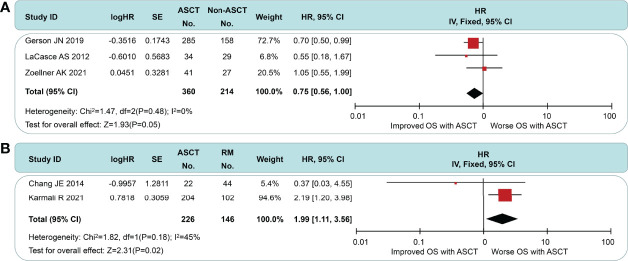
Meta-analysis results of overall survival: **(A)** Subgroup analysis for patients with non-intensive induction. **(B)** Survival analysis between patients undergoing autologous stem cell transplantation and rituximab.

All of the included studies had available OS data (**Figure 5**), and a pooled analysis showed a significant OS benefit with the ASCT approach (HR = 0.77, 95% CI 0.65 to 0.92). Among-group dissimilarities differed only weakly (*I* ^2^ = 29%, *p* = 0.21). In the subgroup comparison of non-intensive induction therapy (**Figure 6A**), three studies were pooled, and no OS differences were observed (HR = 0.75, 95% CI 0.56 to 1.00). *I* ^2^ value was 0 (*p* = 0.21), which suggested a low degree of heterogeneity. In the intensive induction subgroup analysis, only one article was for eligibility. The analytic sample consisted of 454 individuals: 307 (67.6%) in the ASCT group and 147 (32.4%) in the control group. The OS of both groups did not exhibit a significant difference (HR = 0.92, 95% CI 0.61 to 1.40). Similarly, we performed the subgroup analyses of OS between ASCT and rituximab arms (**Figure 6B**). The ASCT group had poorer OS than the rituximab group (HR = 1.99, 95% CI 1.11 to 3.56), with acceptable heterogeneity (*I* ^2^ = 45%, *p* = 0.18).

## Discussion

Current first-line treatments were chemo-immunotherapy regimens containing rituximab and HiDAC followed by ASCT for young and fit MCL pts. However, the only evidence supporting ASCT is from the pre-rituximab and non-HiDAC era. Recent data of 14-year follow-up of this RCT showed a lack of significant PFS and OS benefit for pts who received rituximab plus ASCT (14). Due to the lack of new RCTs, whether ASCT is still the modality of choice in MCL becomes a matter of debate in the rituximab plus HiDAC era. To our knowledge, this was the first meta-analysis assessing the therapeutic value of early ASCT in newly diagnosed transplant-eligible MCL pts across studies worldwide.

We must emphasize three critical aspects of this study design. First, we excluded pts without rituximab induction. A real-world observational study from the Nordic Lymphoma Group proposed that rituximab can significantly improve the prognosis of MCL (15). More retrospective and prospective data have confirmed this view (19–21). To avoid the impact of the absence of rituximab on survival, we specified a limit for induction strategy. Second, we carried out subgroup analyses based on the intensity of induction regimens. Due to the small sample size, augmented intensity induction treatments are not restricted to HiDAC-containing regimens. So far, no prospective studies were using intensive induction to compare ASCT consolidation with other post-induction strategies. Last, we compared early ASCT consolidation with post-induction RM instead of ASCT. Previous studies looked toward RM after ASCT in young and fit pts or RM without ASCT in elder and unfit pts (19, 22, 23). There was no attempt to systematically analyze the possibility of ablating ASCT for transplant-eligible MCL in the front-line setting.

In this study, we observed that the consolidation efficacy of ASCT was related to the intensity of induction regimens. ASCT consolidation could provide a significant PFS benefit for pts with non-intensive therapy but an attenuated PFS benefit for pts using intensive induction regimens. For OS, ASCT had no benefit regardless of induction intensity. A likely explanation is that ASCT is a distinct form of high-intensity chemotherapy, which could delay disease progression to some extent but not improve long-term survival. Based on the study by Zoellner et al. (14), we know that relapse after ASCT is inevitable. Increased risk of long-term adverse events and secondary malignancies associated with ASCT were also concerns in clinical practice. A study by LaCasce et al. (13) showed that rates of febrile neutropenia (44%) and hospitalization caused by complications of therapy (75%) were numerically highest in the R-hyper-CVAD+ASCT therapy group compared to the non-ASCT group. In the study by Gerson et al. (16), at a median follow-up of 76.8 months, 2.5% (*n* = 16) of pts who underwent consolidative ASCT developed secondary myelodysplastic syndrome or acute myeloid leukemia, although no statistical differences were found when compared with those treated without ASCT (2.5% vs. 1.3%, *p* = 0.36). Despite a lack of sufficient comparable data, we still expect alternative ways with more promising efficacy and lower toxicity eagerly. Subgroup analysis showed both PFS and OS benefits in favor of RM than ASCT, similar to Vidal et al.’s conclusions (24). Hilal et al. (23) demonstrated that for MCL pts who responded to induction chemotherapy and underwent ASCT consolidation, RM therapy after transplantation improved PFS and OS. Despite some variability in pts’ characteristics, RM is beneficial in most trials. Kluin-Nelemans et al. (19) found that RM obtained benefits after R-CHOP but not after FCR. In the present analysis, we cannot explore the relationship between the effect of RM and induction regimens because of the small sample size.

The specific scheme of RM therapy may be another concern. In the trial by Chang et al. (5), RM was administered at 375 mg/m^2^ weekly × 4 every 6 months for 16 doses, to be initiated 4 to 8 weeks after completion of chemotherapy. In the trial by Tessoulin et al. (22), RM was administered as a single dose of 375 mg/m^2^/day every 2 months for 3 years. In one meta-analysis of 7 such studies, there are many other similar protocols for RM therapy. In short, regarding the optimal RM strategy, the study is inconclusive. There is still much work to be done in the future.

Besides RM, other maintenance strategies such as bortezomib, lenalidomide, and ibrutinib are under investigation as well (25–29). The comparison between studies 50403 and 59909 with long-term follow-up suggests a PFS benefit from the addition of bortezomib post-transplant (25). The SWOG S0601 trial of R-CHOP with concurrent and maintenance bortezomib suggested a PFS benefit in 65 non-transplant MCL pts (26). Another randomized phase III trial of transplant-ineligible MCL pts showed that VR-CAP improved PFS and OS than R-CHOP, whose benefits may be due to the use of bortezomib during induction or maintenance or both (27). The Italian Lymphoma Foundation recently showed that lenalidomide maintenance after ASCT can improve PFS in MCL pts (28). Triangle, an ongoing randomized phase III trial (EudraCT-no. 2014-001363-12), randomly allocated young, fit pts newly diagnosed with MCL into three arms: (i) an alternating R-CHOP/R-DHAP induction followed by myeloablative consolidation (arm A); (ii) ibrutinib is added to the R-CHOP cycles and applied as maintenance for 2 years (arm A+I); and (iii) the same induction and maintenance are applied, but high-dose consolidation and ASCT are skipped (arm I). As of July 30, 511 of up to 870 pts have been randomized from 12 different European countries (29). We look forward to the results of Triangle, especially for arm I. Long-term combined results of two trials implementing R-MACLO-IVAM induction followed by thalidomide or RM in 44 untreated MCL pts indicated that R-MACLO-IVAM followed by maintenance therapy is an effective regimen to induce long-term remission in MCL without the need for consolidation with ASCT (30). Accumulative evidence suggests that a paradigm shift is occurring in the treatment of newly diagnosed MCL pts in the era of the new drug, suggesting the possibility of using new drug maintenance as an alternative treatment. The optimal maintenance agent type, dose, and duration of MCL should be explored in future clinical investigations.

Minimal residual disease (MRD) has shown the prognosis value in MCL (21, 31). One meta-analysis indicated that MRD positivity after induction and consolidation treatments was associated with worse PFS and OS for MCL. Tan et al. (32) reported that MCL pts achieving MRD negativity after induction therapy with R-hyper-CVAD have excellent long-term outcomes and may reasonably avoid consolidative ASCT despite its small size. In the MRD analysis by Callanan et al. (33), RM provides longer PFS and OS regardless of MRD status pre- and post-ASCT in MCL pts who completed R-DHAP induction therapy. Sequential MRD monitoring during treatment offers strong potential for early clinical outcome prediction, as a surrogate clinical endpoint, and for MRD-guided, risk-adapted treatment in MCL.

Some limitations are worth mentioning. First, the number of included studies was too small to assess the potential publication bias. Second, only one RCT met the inclusion studies due to the low incidence of MCL. In the future, our conclusions still need to be verified by large-sample, multi-center, and more rigorously designed RCTs.

## Conclusions

In the rituximab plus HiDAC era, the benefit of ASCT as a component of first-line treatment has been weakened. RM therapy may have a potential to be used as an alternative to conventional ASCT. These are, of course, speculative based on the current dataset; thus, our meta-analysis cannot provide firm advice on this matter. As more and more clinical trials are ongoing, the challenge in the treatment of MCL will be how to use the best treatment combinations to reach the following goals: low recurrence rate, fewer adverse events, and long-term survival.

## Data Availability Statement

The original contributions presented in the study are included in the article/**Supplementary Material**. Further inquiries can be directed to the corresponding authors.

## Author Contributions

HL: Conceptualization, data curation, validation, writing—original draft, and visualization. XS: Methodology, data curation, and writing—original draft. HF: Software, investigation, data curation, and writing—original draft. LC: Resources, methodology, and writing—review and editing. YM: Methodology, validation, and writing—review and editing. XZ: Investigation, resources, and writing—review and editing. WW: Validation, visualization, and writing—review and editing. WX: Conceptualization, methodology, and writing—review and editing. JL: Conceptualization, writing—review and editing, and project administration. LF: Conceptualization, writing—review and editing, supervision, and funding acquisition. All authors contributed to the article and approved the submitted version.

## Funding

This study was supported by grants from the National Natural Science Foundation of China (81720108002), the National Science and Technology Major Project (2018ZX09734-007), the Translational Research Grant of NCRCH (2020ZKZB01), and the CSCO Research Foundation (Y-Roche2019/2-0090).

## Conflict of Interest

The authors declare that the research was conducted in the absence of any commercial or financial relationships that could be construed as a potential conflict of interest.

## Publisher’s Note

All claims expressed in this article are solely those of the authors and do not necessarily represent those of their affiliated organizations, or those of the publisher, the editors and the reviewers. Any product that may be evaluated in this article, or claim that may be made by its manufacturer, is not guaranteed or endorsed by the publisher.
