# Supplementary material for: First-Line Autologous Stem Cell Transplantation for Mantle Cell Lymphoma: A Systematic Analysis and Treatment Recommendation
Source: Front Oncol. 2022 May 11;12:881346. doi: 10.3389/fonc.2022.881346 (PMC9130771; doi:10.3389/fonc.2022.881346)
Supplement: Supplementary file 1 [file DataSheet_1.docx]

Supplementary Material

# **Supplemental file 1: Detailed search strategy.** This document describes the search strategy in details.

We will search the following electronic databases: PubMed, MEDLINE, Embase, Cochrane Library. There will be no restriction on language of publication. We will search additional studies in the reference lists of all identified publications, including relevant meta-analyses and systematic reviews.

1. **PubMed:**

Search using the NCBI interface from the earliest available date of indexing through September 15, 2021.

| # | Query | Results |
| --- | --- | --- |
| 1 | (((((Lymphoma, Mantle-Cell[MeSH Terms]) OR (Lymphoma, Mantle Cell)) OR (Lymphomas, Mantle-Cell)) OR (Mantle-Cell Lymphoma)) OR (Mantle Cell Lymphoma)) OR (MCL) | 15,739 |
| 2 | ((((((((Transplantation, Autologous[MeSH Terms]) OR (Autotransplantation)) OR (Autografting)) OR (Autologous Transplantation)) OR (Transplantations, Autologous)) OR (ASCT)) OR (AHCT)) OR (autologous stem cell rescue)) OR (transplantation) | 928,045 |
| 3 | Survival | 2,298,300 |
| 4 | 1 AND 2 AND 3 | 770 |

1. **MEDLINE**

Search using the Ovid interface from the earliest available date of indexing through September 15, 2021.

| # | Query | Results |
| --- | --- | --- |
| 1 | Lymphoma, Mantle-Cell.sh. or Lymphoma, Mantle Cell.af. or Lymphomas, Mantle-Cell.af. or Mantle-Cell Lymphoma.af. or Mantle Cell Lymphoma.af. or MCL.af. | 15,038 |
| 2 | Transplantation, Autologous.sh. or Autotransplantation.af. or Autografting.af. or Autologous Transplantation.af. or Transplantations, Autologous.af. or ASCT.af. or AHCT.af. or autologous stem cell rescue.af. or transplantation.af. | 828,954 |
| 3 | Survival.af. | 1,348,650 |
| 4 | 1 and 2 and 3 | 606 |

1. **Embase**

Search using the Elsevier interface from the earliest available date of indexing through September 15, 2021.

| # | Query | Results |
| --- | --- | --- |
| 1 | mantle AND cell AND lymphoma OR (lymphoma, AND mantle AND cell) OR (lymphoma, AND 'mantle cell') OR (mantle AND cell AND lymphoma AND mcl) OR ('mantle cell' AND lymphoma) OR (mcl AND mantle AND cell AND lymphoma) | 15,038 |
| 2 | autologous AND hematopoietic AND stem AND cell AND transplantation OR ('auto hsc' AND transplantation) OR 'auto hsct' OR (autologous AND haematopoietic AND stem AND cell AND transplantation) OR (autologous AND hematopoietic AND stem AND cell AND hsc AND transplantation) OR (hsc AND transplantation; AND autologous AND hsct) OR (autologous AND hscts) | 22,173 |
| 3 | survival | 1,920,471 |
| 4 | #1 AND #2 AND #3 | 1,962 |

1. **Cochrane Library**

Searched using the Wiley interface from the earliest available date of indexing through September 15, 2021.

| # | Query | Results |
| --- | --- | --- |
| 1 | Lymphoma, Mantle-Cell | 667 |
| 2 | Lymphoma, Mantle Cell | 679 |
| 3 | Lymphomas, Mantle-Cell | 116 |
| 4 | Mantle-Cell Lymphoma | 667 |
| 5 | Mantle Cell Lymphoma | 679 |
| 6 | MCL | 716 |
| 7 | #1 or #2 or #3 or #4 or #5 or #6 | 1,020 |
| 8 | Transplantation, Autologous | 6,497 |
| 9 | Autotransplantation | 519 |
| 10 | Autografting | 201 |
| 11 | Autologous Transplantation | 6,497 |
| 12 | Transplantations, Autologous | 48 |
| 13 | ASCT | 1,291 |
| 14 | AHCT | 34 |
| 15 | autologous stem cell rescue | 212 |
| 16 | transplantation | 40,144 |
| 17 | #8 or #9 or #10 or #11 or #12 or #13 or #14 or #15 or #16 | 40,715 |
| 18 | survival | 114,828 |
| 19 | #7 and #17 and #18 | 178 |

**
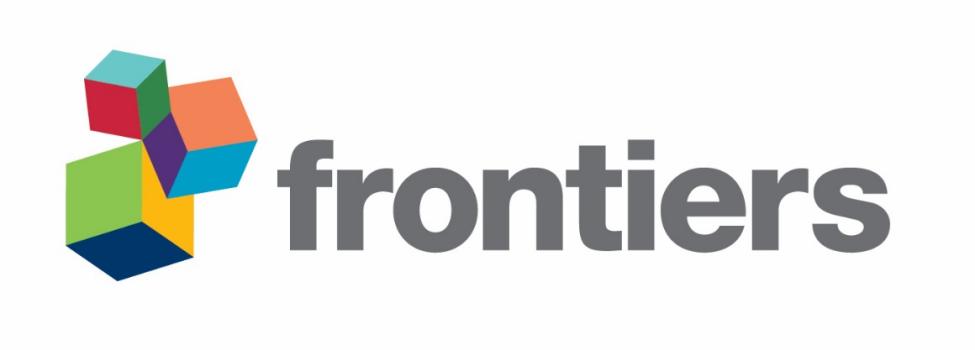
**
